# Histology and External Morphological Assessment on Ovarian Maturation Stages of Wild Female Banana Shrimp, *Penaeus merguiensis* (de Man, 1888) for Broodstock Selection Purpose

**DOI:** 10.21315/tlsr2022.33.1.6

**Published:** 2022-03-31

**Authors:** Hidayah Manan, Adnan Amin-Safwan, Irma Wahida Shakri, Nor Azman Kasan, Mhd Ikhwanuddin

**Affiliations:** Higher Institution Centre of Excellent (HICoE), Institute of Tropical Aquaculture and Fisheries, Universiti Malaysia Terengganu, 21030 Kuala Nerus, Terengganu, Malaysia

**Keywords:** Banana Shrimp, Broodstock Selection, Oocyte, Ovarian Maturation Stage, *Penaeus merguiensis*, Yolky Globules, Udang Pisang, Pemilihan Induk, Oosit, Peringkat Kematangan Ovari, *Penaeus merguiensis*, Globul Kuning

## Abstract

Study on ovarian maturation stages were carried out using external morphological assessment of ovarian colourations and histological assessment of Oocyte structure for broodstock selection purpose. Stage 1 to Stage 4 of female shrimps broodstock were sampled from Kuala Muda, Kedah, Malaysia. Four obvious colourations of ovary stages were identified which are: yellow (Stage 1), green yellowish (Stage 2), light greenish (Stage 3) and dark green (Stage 4) through the morphological assessment. The Gonadosomatic Index (GSI) showed significant increment as the gonad reach advance stages. Histological assessment of Stage 1 ovary identified perinucleolar oocyte (PO) with small size oocytes. Stage 2 ovary was identified with yolkless oocyte (YO), Stage 3 ovary was identified with late stage of yolky globules and Stage 4 ovary was identified with fully yolky globules. From the results achieved, it can be concluded that the ovarian colourations and GSI can be applied to identify the ovarian maturation stages, meanwhile, the histology assessment is the most precise method to determine the ovarian maturation stages in more details and accurate for each of the ovarian maturation stages.

## Abstract (fr)

Kajian mengenai peringkat kematangan ovari telah dijalankan menggunakan penilaian morfologi luaran pewarnaan ovari dan penilaian histologi struktur oosit untuk tujuan pemilihan induk. Peringkat 1 hingga Peringkat 4 induk udang betina telah diambil sampel dari Kuala Muda, Kedah, Malaysia. Empat warna jelas peringkat ovari telah dikenal pasti iaitu: kuning (Peringkat 1), hijau kekuningan (Peringkat 2), hijau muda (Peringkat 3) dan hijau gelap (Peringkat 4) melalui penilaian morfologi. Indeks Gonadosomatic (GSI) menunjukkan peningkatan yang ketara apabila gonad mencapai peringkat lebih tinggi. Penilaian histologi ovari Peringkat 1 mengenal pasti perinucleolar oocyte (PO) dengan oosit saiz kecil. Ovari Peringkat 2 telah dikenal pasti dengan yolkless oocyte (YO), ovari Peringkat 3 telah dikenal pasti dengan peringkat akhir globul kuning dan ovari Peringkat 4 telah dikenal pasti dengan globul kuning sepenuhnya. Dari keputusan yang dicapai, dapat disimpulkan bahawa pewarnaan ovari dan GSI boleh digunakan untuk mengenal pasti peringkat kematangan ovari, sementara itu, penilaian histologi merupakan kaedah yang paling tepat untuk menentukan peringkat kematangan ovari dengan lebih terperinci dan tepat untuk setiap peringkat.

**Highlight**

- Maturation stages were identified; yellow (Stage 1), green yellow (Stage 2), light green (Stage 3) and dark green (Stage 4).
- A histological assessment of ovarian maturation stages was really valuable in the identification of the ovary development for female shrimp broodstock.
- Additional knowledge generates on the ovarian maturation stages in the broodstock management and selection technique in aquaculture.

## INTRODUCTION

Banana shrimp, *Penaeus merguiensis* is one of the most valuable penaeid shrimp in the marine crustacean group (Powell *et al.* 2015). This species has become one of the most important commercialise species of shrimp in Australia, New Guinea, Indonesia, Malaysia, India and to Arabian Gulf (Phongdara *et al.* 1999; Hoang *et al.* 2002; Hidayah *et al.* 2012; Ikhwanuddin *et al.* 2019). Due to the higher commercially prices of tiger shrimp, *P. monodon* and *P. merguiensis* in local markets, Malaysia has stepped up and become one of the leading tropical countries in shrimp aquaculture industry (Iehata *et al.* 2017). Due to it higher demand, worldwide efforts have been made up to develop the secure captive breeding programs of *P. merguiensis* in order to mitigate and reducing the risks of relying on the wild broodstock. Nguyen *et al.* (2014) had identified the large scale of aquaculture production of *P. merguiensis* has been carry out in Southeast Asia and in Australia. However, there is very little knowledge and scarce information regarding *P. merguiensis*’s breeding, physiology and immunity (Wu *et al.* 2019).

Reproductive biology is important for the proper management of fisheries (Perdichizzi *et al*. 2012; Amin-Safwan *et al.* 2018). Oocyte maturation and ovarian development are continually event that is essential in reproduction system (Ruan *et al.* 2020). Thus, most of the penaeid hatcheries have gain multiple efforts to ensure reproductive success of the prawn broodstock focusing on the maturation stage of the spawned female (Alfaro 1993; Kannan *et al.* 2015). Shrimp farmers always depend on the wild stocks of penaeid shrimp for the larvae production in present practice. However, the fluctuation of the availability of the wild broodstock has reduced the hatchery production (Kannan *et al.* 2014). Throughout the world, there are number of studies have been conducted on the ovarian development of penaeid shrimp; however, there are just based on the colouration of the ovaries, with histological stage changes on the ovaries was very few been studied. Histological analysis it was proved increase the precision in measuring the Oocyte size during the ovarian maturation and development stages in the Penaeids shrimp (Crisp *et al.* 2017). Based on previous studies, the histological analysis on the ovarian maturation provides more reliable data about the spawning and breeding of the shrimp (Ayub & Ahmed 2002). Since year ago, the Department of Fisheries Malaysia actively encouraging the young farmers to have interest in culturing the *P. merguiensis* species (Othman 2008), thus proved this species as the targeted and demanded aquaculture species.

The details study on the biological of penaeids shrimp is still require and inadequate information and data on the reproduction, spawning seasons and maturation of these shrimps remains scanty (Hendry *et al.* 2019). Indeed, due to the important of culturing this *P. merguiensis* species and the important on the knowledge of the broodstock maturation, management and selection purpose, therefore this study was carried out to assess the ovarian maturation stages of wild caught female broodstock started for Stage 1 until Stage 4, for broodstock selection purpose. Results from the previous studies were carried out based on the external morphology (ovarian colouration) for the identification of ovarian stage instead of histology analysis that gives more precise results. Thus, this study really useful to be applied in identifying the ovarian maturation stages of female broodstock. Ultimately, this study can also be used to identify the reproductive biological change during maturation stages for breeding programs of any marine shrimp broodstock in the future.

## METHODOLOGY

### Sampling and Study Location

**Figure 1:**
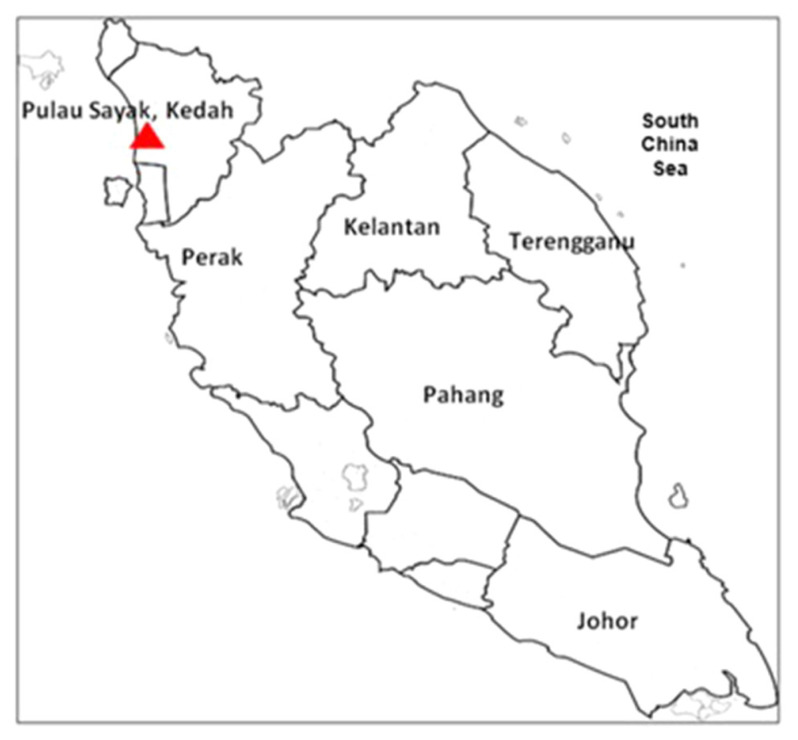
Sampling location of female banana shrimp, *P. merguiensis* from Pulau Sayak, Kedah, Malaysia coastal water. Latitude: longitude ((5°40′34.92″N 100°24′04.08″E)

Female shrimp, *P. merguiensis* comprised of three samples for each ovary stages with body weight, BW > 20 g were sampled from Pulau Sayak, Kedah, Malaysia (5°40′34.92″ N 100°24′04.08″ E) (Fig. 1). The sampling was conducted in September to December by weekly sampling). Trammel net consists of 3-layer net was used to catch the shrimp samples. The shrimp body weight (BW), carapace length (CL), and total length (TL) were measured using electronic balance (Shimadzu; 0.001 g) and also with digital Vernier caliper (0.001 cm), respectively. Gonad maturation stages were selected based on the size of the external diameter of the gonad at the top body of the female shrimp. The Stage 5 (post gonad stage) or the gonad stage after the spawning was not included in the case study due to our aimed to focus only on the Stage 1 until Stage 4 of the maturation stages of the shrimp’s gonad. Samples were undergoing dissection for ovarian colouration and Gonadosomatic Index (GSI) determination after the morphometric data were measured. The small lobes of ovary were then fixed in Davidson’s solution and transported back to Universiti Malaysia Terengganu (UMT) for further histological analysis.

### Ovarian Colourations

The ovarian maturation stages were distinguished by the colouration of the ovary based on observations from previous studies by Ayub and Ahmed (2002) and Kannan *et al.* (2014), where Stage 1 = yellow, Stage 2 = green-yellow, Stage 3 = light green and Stage 4 = dark green in colour. The external morphology of the ovarian stage identification was carried out based on the colouration of each ovary stage prior to the histological analysis for each ovary stages.

### Histological Analysis

The small lobes of ovary were fixed in the Davidson’s solution for 24 h (Amin-Safwan *et al*. 2018). The ovary was dissected and placed in histology cassette, then followed by placement in 70% alcohol overnight. Samples were transferred into the tissue processor on the next day. The procedure of tissue processing followed the standard protocol which were; alcohol 70% for 1 h, continued by alcohol 90%, alcohol 95%, alcohol 100%, (xylene I, xylene II, xylene III) for clearing and wax (2 h) continued with another wax (2 h) for impregnation (total up to 18 ± 1 h). The samples were embedded, cut and sectioned in 5 μm size. Sectioned samples were dried on a hot plate (40ºC) overnight and continued with staining procedure. The samples were stained using hematoxylin and eosin (H&E) standard protocol as followed: xylene I (5 min), continued by xylene II (5 min), 100% alcohol I (5 min), 100% alcohol II (5 min), 95% alcohol (2 min), 70% alcohol (2 min), running tap water (2 min), hematoxylin (10 min), running tap water (2 min), acetone acid (3 dip), running tap water (2 min), 2% potassium acetate (3 min), running tap water (2 min), eosin (5 min), 95% alcohol (5 min), 95% alcohol (5 min), xylene I (5 min), xylene II (5 min) and finished with DPX mounting. Oocyte structures were analysed under advanced microscope (*Nikon eclipse* 80i) and labeled for it ovarian maturation stages for further data analysis.

### Data Analysis

The GSI was calculated based on the ovary weight and total body weight (Amin-Safwan *et al*., 2016; 2018) of the female shrimp broodstock. The formula for GSI was calculated as followed:


$$\text{GSI}=\frac{\text{Ovary weight }(\text{g})}{\text{Total body weight }(\text{g})}\times 100$$

One-way ANOVA (SPSS version 20.0) with post-hoc and Tukey test was carried out to identify the significant difference (*p* < 0.05) in the GSI value according to respective stages (Stage 1, Stage 2, Stage 3 and Stage 4). The data were presented as mean ± standard deviation.

## RESULTS

### External Morphology (Ovarian Colourations and GSI)

Table 1 showed the details description on the BW, CL, TL and ovary weight for each ovarian maturation stage identified. For Stage 1, the mean weight was 7.66 ± 0.11 g, Stage 2 was 8.40 ± 0.4 g, Stage 3 with 8.80 ± 0.2 g and Stage 4 about 9.56 ± 0.55g. There was an increment of the ovary weight when it reaches advance stages.

**Figure 2:**
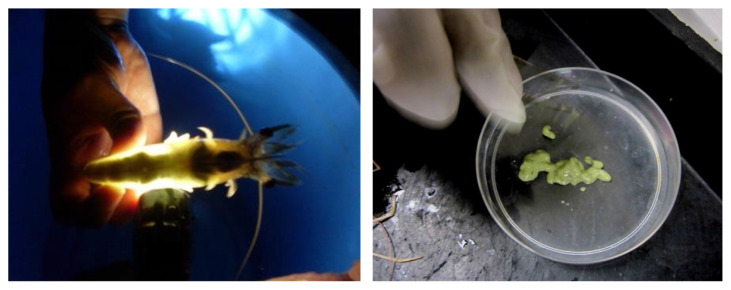
Ovary Stage 4 checked on top of the shrimp body appeared in green colour after dissected from matured female shrimp, *P. merguiensis*

Through the ovary external morphology identification, there were four obvious colourations of the ovarian maturation stages, which were: Stage 1 (yellow), Stage 2 (green-yellow), Stage 3 (light green) and Stage 4 (dark green). The ovary which in Stage 4 (dark green) was clearly visible through the top body of the *P. merguiensis*. Fig. 2 showed the process to check on the ripen stage on the top of the shrimp body, and also the colourations of the ovary after dissected out from Stage 4 of the matured female *P. merguiensis*.

**Figure 3:**
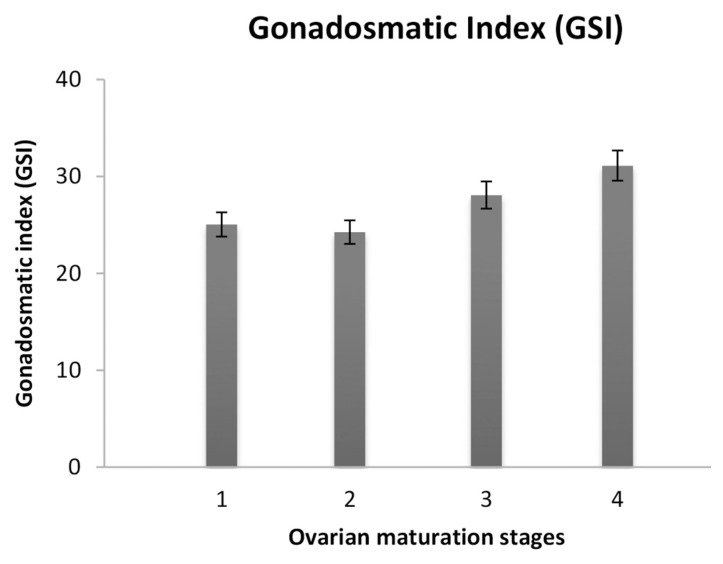
GSI for Stage 1 until Stage 4 of ovarian maturation stages of female *P. merguiensis* broodstock.

In Fig. 3, the GSI for each stage of the ovarian maturation of female broodstock was presented. It was identified that in Stage 1, the mean GSI was 25.04 ± 1.065, GSI for Stage 2 was 24.25 ± 0.984, Stage 3 was 28.08 ± 3.003 and in Stage 4, the GSI recorded was 31.11 ± 1.993. The GSI value was increase as reached advances stages. There was significant difference (*p* = 0.009; *p* < 0.05) between the GSI to respective stages.

### Histological Assessment

**Figure 4:**
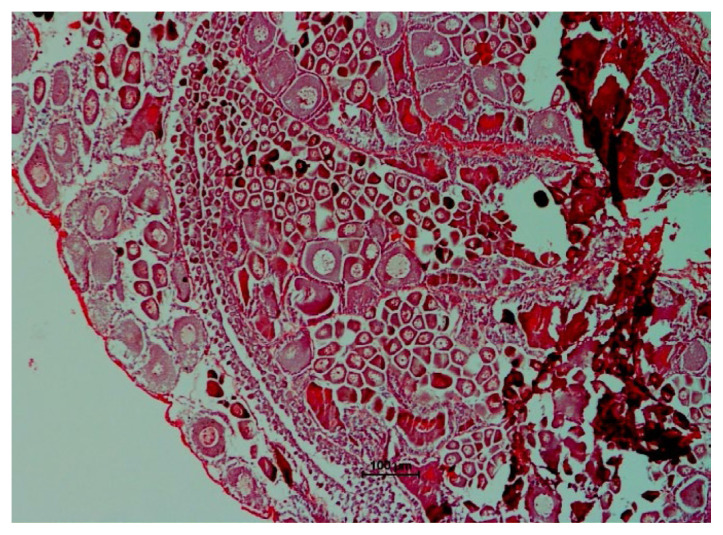
Histological analysis for immature Stage 1 ovary. Ovary was identified with PO and small size of oocytes. The ovary was identified yellow in colour after dissection.

**Figure 5:**
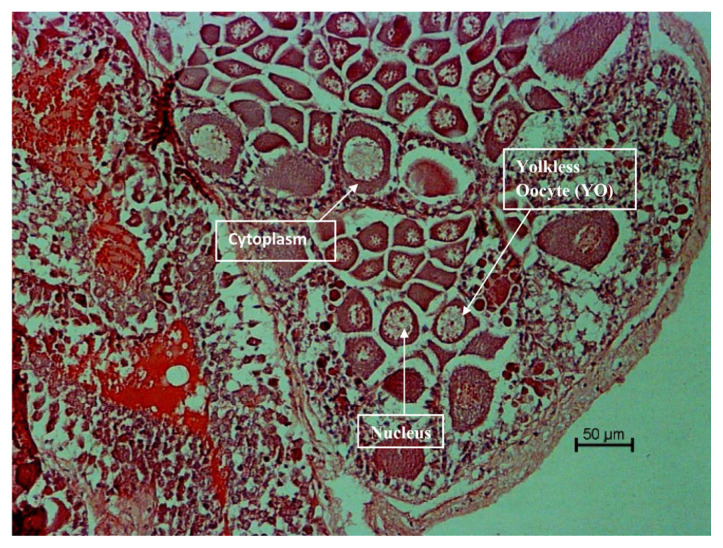
Histological analysis for early mature Stage 2 ovary. Ovary was identified with YO, increase of nucleus size and decreased of cytoplasm size. Ovary was identified with green yellow in colour after dissection.

**Figure 6:**
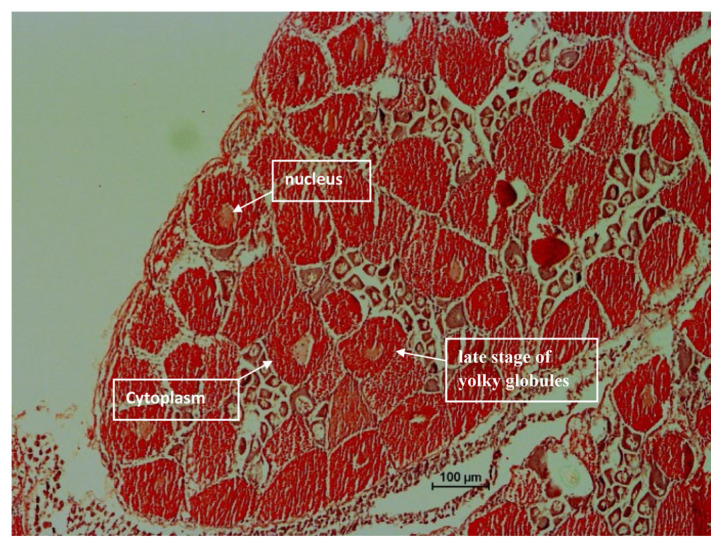
Histological analysis for late matured Stage 3 ovary. The ovary was identified with late stage of yolky globules, decrease of nucleus size and increase of the cytoplasm size. The ovary was identified with light green in colour after dissection.

**Figure 7:**
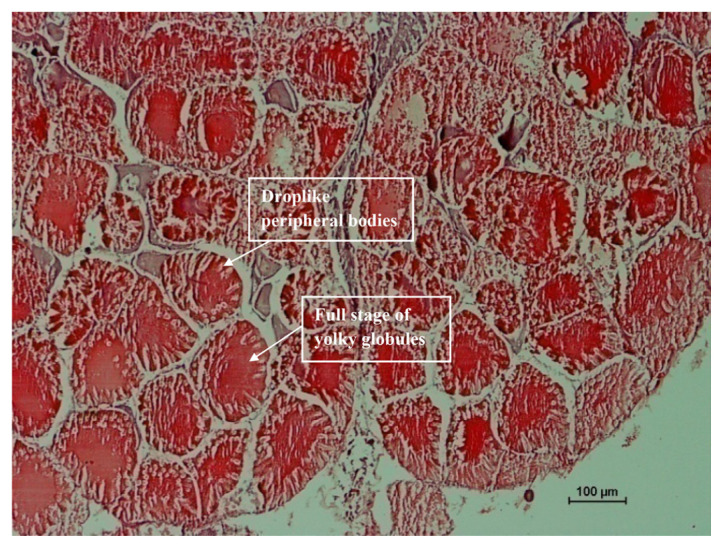
Histological analysis for fully matured Stage 4 ovary. Ovary was identified with fully yolky globules, appearance of droplike peripheral bodies in the ovary sectioning. Ovary was identified with dark green in colour after dissection.

The histological analysis was carried out for each ovarian maturation stage of *P. merguiensis*. For the immature Stage 1 ovary, the perinucleolar oocyte (PO) and small size oocytes were identified from the sectioning of ovary (Fig. 4). In the early mature of Stage 2, yolkless oocyte (YO) was identified with size of nucleus identified greater than cytoplasmic size (Fig. 5). In late matured Stage 3, the late stage of yolky globules was presented and identified with cytoplasmic size increased (Fig. 6). The nucleus size started decreased in Stage 3 ovary. In fully matured Stage 4 ovary, the full stage of yolky globules was identified with appearances of drop-like peripheral bodies presented (Fig. 7). Details description on the ovarian assessment with the colourations for each ovarian stage was described in Table 2.

## DISCUSSION

The appearance of different type of oocytes which observed during histological sectioning is similar with the identification reported by Ayub and Ahmed (2002) in *M. affinis*, which described the ovarian development stage in shrimp from the coast of Pakistan. The other classical study conducted on the histological observation of the ovaries of shrimp also identified five distinct ovarian developmental changes similar with identified in *L. setiferus* (King 1948) and in *P. brasiliensis* (Latreille 1817). The oocytes can be identified in PO, YO and yolky oocytes in the present study. According to Crisp *et al.* (2017), the external characteristic of the ovary such as colour, size and texture tissue are most related to development of the internal organs of the germ cells. In the present study it can be identified that the size of the cytoplasmic in Stage 3 were larger than in size of cytoplasmic identified in Stage 2. The size of the nucleus becomes smaller due to the development size of the cytoplasmic in the Stage 3 ovary cells as the oocytes become yolky during ripen. The colouration of the external ovary stage in the present study also less similar with the identification done by Ayub and Ahmed (2002), and Kannan *et al.* (2014); which identified yellow in Stage 1, green-yellow in Stage 2, light green in Stage 3 and dark green in Stage 4 of ovarian colouration changes. The yellow colour of the ovaries is mostly near to ripe stage and the light green and dark green colour is nearly ripe and fully ripe stage of the ovarian maturation which is easier to distinguish from the exoskeleton part of the body (Ayub & Ahmed 2002; Tuma 1967; Quintero & Gracia 1998; Crisp *et al.* 2017). It also creates a confusion between determining the Stage 3 and Stage 4 because the ovary will developed in whole cephalothoracic cavity also with different shades of green colouration.

Previous study conducted identified that the early ripe which was Stage 3 and the ripe stage which was Stage 4 females can be reliably separated only by identification through histological ovary examination (Crisp *et al.* 2017). From the previous study, they included the spent stage in the maturation stage of shrimp, which made in have five stages of ovarian maturation stages (Kannan *et al*. 2014). However, in this study, we identified four stages which did not include the spent stage as no spent stages shrimps was caught or recorded during the sampling. This is similar results as previous study by Ohtomi *et al.* (1998) that identified four development stages in mud shrimp, *Solenocera melantho* which were; quiescent, developing, early ripe and ripe. In this present study, the GSI value increasing from 25.04 (Stage 1) to 31.11 (Stage 4) showed that precise development (*p* = 0.009; *p* < 0.05) of the ovarian maturation stages of the female broodstock which is also less similar with the studied done by Crisp *et al.* (2017) that identified increase of GSI value on the studied shrimp ovaries. The development of the colour, size and textures of the ovary is related closely to the development of the ovaries (Quintero & Gracia 1998). Therefore, this histological analysis is valuable for the identification of the ovarian maturation stage for the broodstock selection of *P. merguiensis* shrimp and also can be practically utilised to identify other type of penaeid marine shrimp.

## CONCLUSION

There are four stages of ovarian maturation for female broodstock that have been successfully identified from the sample analyses conducted. From the result achieved, there are obviously series of colour changes from Stage 1 to Stage 4 during the ovarian development. Throughout the histological analysis, the PO and small size oocytes were identified in Stage 1, YO was identified in Stage 2 and the late stage of yolky globules was identified in Stage 3, and fully yolky globules was identified in Stage 4 from the sectioning part of the ovaries. Stage 5 (post spawned or post gonad stage) shrimp cannot be reached during the sampling from wild. The results achieve through histology analysis is more reliable as the reproductive biology can be recognised and identified instead of the colourations of each stage of the ovary respectively. This study was really helpful in determining the ovarian maturation stages and can be referred for broodstock management and selection purpose in the future.

## Figures and Tables

**Table 1:** Details description on the body weight, total length, ovary weight for each ovarian maturation stages of female shrimp *P. merguiensis.*

Ovarian maturation stages	No. of shrimps	Body weight (g)	Carapace length (cm)	Total length (cm)	Ovary weight (g)
1	1	31.9	6.1	15.3	7.6
	2	29.7	6.3	13.4	7.6
	3	30.3	6.4	16.1	7.8
2	1	37.1	7.7	16.1	8.8
	2	31.5	5.9	15.3	8.0
	3	35.5	5.6	15.2	8.4
3	1	29.1	6.3	14.9	8.8
	2	29.3	5.9	14.9	8.6
	3	36.5	7.5	17.4	9.0
4	1	33.0	6.4	15.3	10.2
	2	31.8	6.3	13.4	9.3
	3	27.7	5.7	14.8	9.2

**Table 2:** Details description of the ovary through the histological analysis of each stage (Stage 1 until Stage 4) of female *P. merguiensis* broodstock.

Ovarian maturation stage	Ovary colouration	Details description on the ovary
Stage 1	Yellow	PO and small size oocytes were identified.
Stage 2	Green-yellow	YO was identified, size of nucleus greater than cytoplasmic size.
Stage 3	Light green	Late stage of yolky globules was identified, cytoplasmic size increased.
Stage 4	Dark green	Fully yolky globules were identified, droplike peripheral bodies identified.
